# Prefrontal projections to the nucleus reuniens signal behavioral relevance of stimuli during associative learning

**DOI:** 10.1038/s41598-022-15886-0

**Published:** 2022-07-14

**Authors:** Xiaotian Yu, Fasika Jembere, Kaori Takehara-Nishiuchi

**Affiliations:** 1 Department of Cell and Systems Biology, University of Toronto, Toronto, Canada; 2 Human Biology Program, University of Toronto, Toronto, Canada; 3 Department of Psychology, University of Toronto, Toronto, Canada; 4 Collaborative Program in Neuroscience, University of Toronto, Toronto, Canada

**Keywords:** Classical conditioning, Cortex

## Abstract

The nucleus reuniens (RE) is necessary for memories dependent on the interaction between the medial prefrontal cortex (mPFC) and hippocampus (HPC). One example is trace eyeblink conditioning, in which the mPFC exhibits differential activity to neutral conditioned stimuli (CS) depending on their contingency with an aversive unconditioned stimulus (US). To test if this relevancy signal is routed to the RE, we photometrically recorded mPFC axon terminals within the RE and tracked their changes with learning. As a comparison, we measured prefrontal terminal activity in the mediodorsal thalamus (MD), which lacks connectivity with the HPC. In naïve male rats, prefrontal terminals within the RE were not strongly activated by tone or light. As the rats associated one of the stimuli (CS+) with the US, terminals gradually increased their response to the CS+ but not the other stimulus (CS-). In contrast, stimulus-evoked responses of prefrontal terminals within the MD were strong even before conditioning. They also became augmented only to the CS+ in the first conditioning session; however, the degree of activity differentiation did not improve with learning. These findings suggest that associative learning selectively increased mPFC output to the RE, signaling the behavioral relevance of sensory stimuli.

The ability to form associations between environmental cues and salient outcomes is a crucial cognitive process that organisms rely on to adapt and survive. This associative learning process is frequently studied using classical conditioning paradigms. In particular, trace eyeblink conditioning (TEBC) tests the subject’s ability to associate a neutral sensory stimulus (known as the conditioned stimulus [CS]) with a mildly aversive eyelid shock (known as the unconditioned stimulus [US]) that is presented after a brief time interval (known as a trace interval). The inclusion of this temporal delay consequently necessitates the integrity of forebrain regions, including the hippocampus (HPC)^1–3^ and medial prefrontal cortex (mPFC)^4–7^ in addition to the motor circuitry in the cerebellum and brainstem^8,9^. Moreover, the formation of these CS-US stimulus associations is accompanied by the development of selective firing patterns for the associations in the dorsal HPC^10,11^ and mPFC^12–15^. In addition, with learning, the mPFC developed stronger stimulus-evoked theta and gamma-band oscillatory activity^16–18^. Furthermore, mPFC theta band activity becomes temporally coupled with HPC theta band activity^19^. Collectively, these findings suggest that close interaction between the mPFC and HPC is essential for the formation of stimulus associations in TEBC.

There are several anatomical pathways that may support mPFC-HPC interaction. Although monosynaptic projections originate from the ventral HPC to the mPFC, the mPFC lacks monosynaptic excitatory projections back to the HPC^20,21^. Instead, the mPFC may influence HPC neural activity via several multi-synaptic pathways involving intermediary structures. The nucleus reuniens of the midline thalamus (RE) is thought to be one of these intermediary regions^22–24^, as it possesses reciprocal anatomical connections with the mPFC and HPC^25–27^. Previous studies have shown that the RE is instrumental in supporting mPFC-HPC synchronicity^28–30^ and performance in tasks requiring mPFC-HPC interaction, such as passive avoidance^31^, spatial navigation^32,33^, spatial working memory tasks^28,34^, contextual fear conditioning^35–38^, and sequence memory tasks^39^. In particular, the formation of stimulus associations in TEBC was impaired by high-frequency electrical stimulation of the RE during the first two days of conditioning in mice^40^.

Although the aforementioned evidence supports the necessity of the RE for facilitating mPFC-HPC interaction underlying various forms of cognitive processes, it remains unknown as to what kind of information is transmitted within the mPFC-RE-HPC pathway, and more specifically, the degree to which mPFC output is related to the relational and physical features of a stimulus. To address this point, we conducted photometric recordings of calcium dynamics in axons of mPFC neurons terminating within the RE whilst rats underwent TEBC. The usage of TEBC allows for precise control over stimulus timing and contingency, enabling us to differentiate the activity patterns selective for sensory and relational stimulus features, as well as its correlation with task performance. As a comparison, we investigated the learning-related changes in the activity of mPFC axons terminating within the medial dorsal thalamus (MD). The MD is an ideal control based on its projection specificity, as it receives strong monosynaptic projections from the mPFC^41–43^ but does not project to the HPC^44,45^. We found that prefrontal outputs to the RE and MD were selective for stimulus associations; however, only the former augmented its selectivity with learning.

## Materials and methods

### Subjects

A total of 40 male Long-Evans rats (Charles River Laboratories), 77 days old upon arrival, were individually housed in transparent plastic cages in a home colony room and maintained on a 12-h reverse light/dark cycle (dark from 10:00 to 22:00) with free access to food and water. Their weights three weeks after their arrival ranged from 375 to 410 g. The rationale for the usage of only male rats were: (1) sex differences in eyeblink conditioning learning capabilities due to ovarian hormones^46^, and (2) previous studies examining mPFC function and activity in TEBC were conducted exclusively using male rats^7,12,16,17^. Behavioral experiments were performed during the dark cycle. A total of 22 were used in Experiment 1 (pharmacological inactivation of the RE) and 18 rats were used in Experiment 2 (photometry). In Experiment 1, five rats were removed due to mistargeted cannula, leaving 17 rats for behavioral data analysis. In Experiment 2, four rats were removed due to mistargeted optic fibers, and one rat was removed due to breakage of the internal fiber core during behavioral conditioning, this left a total of 13 rats for behavioral and photometric data analysis. All procedures were performed in accordance with the National Institutes of Health Guide for Care and Use of Laboratory Animals (Publication NO. 85–23, revised 1985), the Canadian Council on Animal’s Care, APA ethical standard, the ARRIVE guidelines, and approved by the University of Toronto Animal Care Committee (AUP20011400).

### Surgery

Rats used in Experiment 1 underwent one surgical procedure involving the implantation of an infusion cannula and eye-wires. Rats used in Experiment 2 underwent two surgical procedures, first a viral vector infusion surgery, followed by an optic fiber and eye-wire implantation surgery.

### Viral surgery

Three weeks after their arrival at the facility, rats in Experiment 2 were anesthetized (2.0–2.5% isoflurane by volume in oxygen at a flow rate of 0.8 L/min; Halocarbon Laboratories) and placed in a stereotaxic frame. The skin and tissue above the skull were retracted and one hole was drilled in both hemispheres above the mPFC. A 30G stainless steel infusion needle connected to a micro-syringe (Hamilton) via polyethylene tubing was used to deliver AAV9.CAG.GCaMP6f.WPRE.SV40 (Addgene) at these coordinates: anteroposterior (AP) = + 2.7, mediolateral (ML) = ± 0.55, dorsoventral (DV) = − 3.9 mm from bregma. The viral vector (0.75 μl/hemisphere) was injected at a rate of 0.1 μl/min. The needle was left in place for five minutes after completion of the injection, afterwards, it was retracted to DV = − 3.7 mm and left there for a further five minutes to ensure successful diffusion of the vector. The needle was then slowly retracted, and the incision was sutured. The rats were treated with an analgesic (carprofen, 5 mg/kg, subcutaneous) for 48 h after surgery. Rats were housed in their home cage for two months following the surgery to allow for viral incubation and expression.

### Implantation surgery

For Experiment 1, five weeks after their arrival at the facility, rats were anesthetized with isoflurane and placed in a stereotaxic frame. The skin and tissue above the skull were retracted and a hole was drilled above the RE in the right hemisphere (AP = − 2.1, ML = + 2.0 mm from bregma). A guide cannula (Plastics One) was lowered through the hole targeting the RE and capped with a dummy stylet (AP = − 2.1, ML = + 2.0, DV = − 6.5 mm from bregma at a 15º angle on the midline). The cannula was secured to the skull using stainless screws and dental acrylic. The infusion cannula used to deliver drugs extended 1 mm past the guide cannula tip. Four Teflon-coated stainless-steel wires attached to a connector cap (Plastics One) were implanted subcutaneously in the upper left *orbicularis oculi* (eyelid muscle) to record electromyogram (EMG) activity and deliver a periorbital shock. A stainless-steel ground screw was installed on the right parietal bone and linked to the connector cap. The connector cap and ground screw were secured to the skull with stainless steel screws and dental acrylic. The rats were treated with an analgesic (carprofen, 5 mg/kg, subcutaneous) for 48 h after surgery and left in their home cage for a week to recover.

For Experiment 2, after viral incubation, rats were anesthetized with isoflurane and placed in a stereotaxic frame. The skin above the skull was retracted and holes were drilled bilaterally above either the RE (AP = − 2.1, ML = ± 2.0 mm from bregma) or MD (AP = − 2.6, ML = ± 2.1 mm from bregma). Optic fibers (NA 0.48, core size 400 µm, FP400URT, Thorlabs) bonded to stainless-steel ferrules (Ø2.5 mm, Thorlabs) were implanted targeting either the RE (AP = − 2.1, ML = ± 2.0, DV = − 7.3 mm from bregma at a 15º angle) or MD (AP = − 2.6, ML = ± 2.1, DV = − 6.35 mm from bregma at a 15º angle). The optic fibers were secured to the skull using stainless-steel screws and dental acrylic. Optic fibers were then capped with a protective dust cap (Thorlabs). The connector cap with its four eye-wires and a ground screw was then installed following the same protocol as Experiment 1. Post implantation surgical care was identical to Experiment 1.

### Differential trace eyeblink conditioning—Experiment 1

In Experiment 1 the behavioral paradigm spanned a total of 12 days. The first two days involved habituating the rats to the conditioning chamber and procedures, whilst the following 10 days involved training in version one of a differential paradigm of trace eyeblink conditioning (DTEBC1). During habituation days (H1 to H2), rats were placed inside a cylindrical plastic container (21 cm in diameter) housed inside a sound and light-attenuating chamber, absent any stimuli, for 50 min. Starting from day three rats underwent DTEBC1 wherein they were presented with two different trial types. In the first trial type, a tone conditioned stimulus (TCS+, 100 ms, 2.5 kHz, 85 dB) was paired with a mild eye-lid shock (unconditioned stimulus [US], 100 ms, 100 Hz) separated by a 500 ms stimulus-free interval. In the second trial type, a light conditioned stimulus (LCS-, 100 ms, 50 Hz) was presented by itself. Although we did not counterbalance the stimuli in Experiment 1, we confirmed with Experiment 2 that rats associate the tone and light with the US comparably, regardless of which of the two stimuli was paired with the US. Daily conditioning sessions consisted of a total of 100 trials, with each trial having a 50% chance of either being a TCS+ trial or a LCS- trial. Trial type presentation was randomized, such that the rat did not know which trial would be presented next. The inter-trial intervals were pseudo-randomized between 20 and 40 s with a mean of 30 s. Each conditioning session lasted approximately 50 min.

Stimulus timing and delivery were controlled by a microcomputer (Arduino Mega, Arduino). The tone stimulus was presented via a ceiling-mounted speaker inside the chamber, the light stimulus was presented via a LED installed at eye level on the side of the chamber, and the US, which was delivered to the eyelid via implanted eye wires, was driven by a stimulus isolator (ISO-Flex, A.M.P.I.). The US shock level was initially set at 0.3 mA and was adjusted individually for each rat to induce the unconditioned response (an eyeblink/head-turn), which was monitored through infrared cameras mounted inside the chambers.

The conditioned response (CR) was defined as eyeblink responses elicited during a 200 ms window immediately prior to the US onset. This same time window was used in trials wherein the US was absent. The above parameters were chosen based on our previous work, which showed that over 10 sessions, rats steadily increased the proportion of trials in which they expressed a CR^18,19^. Eyeblink responses were monitored by recording EMG activity from the left upper *orbicularis oculi* muscle through two surgically implanted stainless-steel wires. EMG activity was band-pass filtered between 0.3 and 3.0 kHz, digitized at 6,102 Hz, and stored using a RZ-5 recording system (Tucker-Davis Technologies).

### Differential trace eyeblink conditioning—Experiment 2

In Experiment 2 the behavioral paradigm spanned a total of 13 days. The first two days involved habituating the rats to the conditioning chamber and procedures, the third day involved naïve stimulus response testing, and the following 10 days involved training in version two of a differential paradigm of trace eyeblink conditioning (DTEBC2). Habituation days one and two were identical to that in Experiment 1 except the duration of each session was 75 min. On the third day (Session 0) the rats were placed inside their conditioning chambers for naïve stimulus response testing. Rats were presented with three trial types, type one involved the presentation of a neutral tone stimulus (TS) by itself, type two involved the presentation of a neutral light stimulus (LS) by itself, and type three involved the presentation of the US by itself. Session 0 consisted of 150 total trials, with 50 trials allotted to each type. Starting from day four (sessions one to 10) rats underwent DTEBC2, wherein they were trained in either tone reinforced (five RE and five MD rats) or light reinforced (one RE and two MD rats) conditioning. In tone reinforced conditioning rats were presented with three different trial types. In the first trial type, a neutral tone stimulus was paired with the US, separated by a 500 ms stimulus-free interval (conditioned stimulus plus and US [CS+US]). In the second trial type the same tone stimulus was presented alone (conditioned stimulus plus [CS+]). In the third trial type, a neutral light stimulus was presented alone (conditioned stimulus minus [CS-]). Rats trained in light reinforced conditioning similarly had three trial types; however, stimulus identities were swapped, such that CS+US trials now paired the light with the shock, the CS+ trials were the light presented alone, and the CS- trials were the tone presented alone. Daily conditioning sessions consisted of a total of 150 trials, CS+US trials occurred with 68% probability while CS+ and CS- trials each occurred with 16% probability. For Session 0 and sessions one to 10 each session lasted approximately 75 min, inter-trial intervals and trial type randomization were similar to Experiment 1.

The inclusion of CS alone trials allowed us to examine the photometric signal change evoked purely by the CS absent any contamination from the US evoked signal change. This allowed us to better measure the change in the CS evoked activity as rats formed differential stimulus associations.

### EMG analysis

All analyses were performed using custom code written in MATLAB (version 2021a, Mathworks)^18,19^. For each session per rat, the amplitude of the EMG signal during each 1 ms time bin was calculated by subtracting the minimum signal from the maximum signal during that bin. EMG amplitude was averaged during a 300 ms window before CS onset in each trial. The baseline was set as the median of averaged EMG amplitude plus one standard deviation. EMG activity above the threshold was averaged together during the 300 ms period before CS onset (Pre-Value) and during a 200 ms window before US onset (CR value). The CR value was designed to capture the adaptive blinking responses that occur immediately before US onset. A trial was defined as a CR trial if the CR value was at least five times higher than that of the Pre-Value. Trials in which the Pre-Value exceeded 30% of the threshold were classified as “hyperactive” trials and discarded. The percent of conditioned responses (CR%) in each trial type was the number of CR trials for that trial type, divided by the total number of valid trials for that type. The percent of hyperactive trials was calculated by dividing the total number of trials classified as hyperactive by the total number of trials (Experiment 1: # hyperactive/100, Experiment 2: # hyperactive/150). To visually depict the temporal pattern of EMG activity on specific days, EMG amplitude was first normalized through division with the Pre-Value in each trial. The normalized EMG amplitude for that trial was then averaged across all valid trials of the same trial type in each rat and then across rats for specific days. For Experiment 1, we examined the temporal pattern of EMG activity by calculating the latency to the onset of the CR, as well as the latency to the peak of the CR in each rat during the last session. Only trials in which the rat showed a CR was used for latency analysis. First EMG amplitude in a 1.3 s window starting from 300 ms before CS onset was extracted from each trial. The EMG values were then subtracted by the Pre-Value (EMG-Pre) and the first 300 ms of data was averaged and multiplied by five to generate the CR threshold value. The latency to the onset of the CR was then defined as the first time-point in a 500 ms window after CS termination wherein the EMG-Pre value exceeded the CR threshold value. The latency to the peak of the CR was defined as the time-point during the same 500 ms window wherein the EMG-Pre value was the highest. Finally, the onset and peak latencies in each trial were averaged across all trials of the same type in each rat.

### Intracranial infusions

Rats were given intracranial infusions of their assigned solutions starting from the second day of habituation and through all 10 days of DTEBC1. Rats restrained in a flexible plastic cone were infused 20 min prior to the start of conditioning. The infusion consisted of either a muscimol hydrobromide solution (0.5 mg in 0.5 mL artificial cerebral spinal fluid [aCSF], G019 Sigma-Aldrich), or aCSF. A total of 500 nl was infused over the course of a minute. Afterward, the infusion cannula was left in place for an additional minute before extraction. Drug concentration, infusion volumes, and infusion rate were chosen based on our previous studies, which found muscimol spread to be confined to a maximal range of 1 mm from the cannula tip^47,48^. The muscimol group totaled eight rats whilst the aCSF control group totaled nine.

### Fiber photometry

Photometric recordings of prefrontal terminals within the RE and MD were performed by measuring bulk fluorescence via a single optic fiber through which excitatory light was delivered and emitted fluorescence was captured. A 465 nm LED modulated at 381 Hz was used to stimulate GCaMP6f., emitting a calcium dependent fluorescence. A 405 nm LED modulated at 221 Hz was used to stimulate GCaMP6f. at its excitation isosbestic wavelength, emitting a calcium independent fluorescence. Modulation was performed via a RZ5 recording system (Tucker Davis Technologies). Light from both LEDs was coupled into an integrated fluorescence mini-cube (IFMC, Doric Lenses) which output both light streams into a coupled fiber patch cord (NA 0.48, core size 400 µm, Doric Lenses). The patch cord was then mated with the implanted optic fiber and ferrule assembly using an opaque zirconia sleeve (Thorlabs). The output power of both LEDs measured at the tip of the patch cord was 60 µW (PM100D, Thorlabs). Emitted fluorescence from the animal passed through the patch cord and into the IFMC which was coupled to a femtowatt photoreceiver (Doric Lenses). Output from the photoreceiver was then fed into the RZ5 recording system which demodulated and sampled the output at 1 kHz. Photometric recordings took place on days two through 13 in Experiment 2. Because each session lasted for 75 min, we only turned on the LEDs for a short duration time-locked to stimuli in order to minimize photobleaching within each session. On day two (Hab 2) the LEDs were turned on for nine seconds every 30 s. On day three (Session 0) the LEDs were turned on four seconds before the onset of the TS, LS, and US, and left on for nine seconds in each trial. On days four to 13 (sessions one to 10), the LEDs were turned on four seconds before the onset of the CS and left on for a total of nine seconds in each trial. In the first two rats conditioned, a small amount of artifact was detected upon US delivery during Session 0, this artifact was not seen for the TS nor LS. Congruent with the subsequent analyses of terminal responses towards the CS, we chose to only analyze the activity evoked by the TS and LS in Session 0.

Using custom written MATLAB code fluorescence activity from 405 nm stimulation was used to correct for motion artifact and photobleaching. First, because the fluorescent output became stabilized within two seconds from the LED onset, the 465 nm and 405 nm signals from the last seven seconds of the nine second LED on time were extracted from each trial. The 405 nm signal was then fitted to the 465 nm signal using a least-squares linear fit approach, the ΔF/F was then calculated as: (465 nm signal—fitted 405 nm signal)/fitted 405 nm signal. The resultant ΔF/F values represented calcium activity two seconds before and five seconds after CS onset in each trial. The ΔF/F values were then averaged across all trials sharing the same trial type for that session.

To depict CS evoked calcium activity in prefrontal terminals within the RE or MD, ΔF/F values in a six second window starting from two seconds before CS onset were first grouped based on trial type, values were then averaged across trials in each session**.** For the comparison of ΔF/F responses to different CS types, area under the curve (AUC) values were calculated by summing the trial averaged ΔF/F values in a two second window for each session in each rat. For baseline AUC, the window started two seconds before the onset of the CS, whilst for CS response AUC, the window started from the termination of the CS. AUC values were then averaged across rats in each session.

### Histology

Once behavioral testing concluded, rats were deeply anesthetized with an excess amount of sodium pentobarbital (80 mg/kg, Bimeda). They were first perfused transcardially with chilled 0.9% physiological saline solution, followed by chilled 4% paraformaldehyde (PFA). Brains were extracted and left submerged within 4% PFA at 4 °C for two hours, followed by submersion in phosphate buffered saline—30% sucrose solution (PBS-Suc) for 48 h. Coronal brain slices (45 μm) across the entire anterior–posterior extent of the mPFC, RE, and MD were collected using a cryostat (CM3050S, Leica Biosystems). Tissue sections were stored in tubes filled with tissue storage buffer (PBS-Suc and ethylene glycol solution), all tubes were then stored at − 20 °C. To locate infusion cannula placements, tissue was mounted on a glass slide and stained with cresyl violet. A thin layer of Cytoseal 280 mounting solution (#8311–4, Thermo Fisher Scientific) was applied along with a glass coverslip. Slides were viewed under a light microscope (Leica DM400b). To locate optic fiber placements, tissue was mounted on a glass slide and then sealed using Cytoseal and a glass coverslip. Slides were viewed under an upright fluorescent microscope (Zeiss AxioImager 2.0). Locations of cannulae and optic fibers were then drawn onto plates from the stereotaxic atlas of the rat brain^49^. Only those rats with cannula accurately targeting the RE were used in Experiment 1, and only those rats with an optic fiber accurately targeting the RE or MD were used in Experiment 2.

### Statistical analysis

The sample size for Experiment 1 (pharmacological inactivation of the RE) was based on our previous behavioral studies using TEBC^7,16,17^. Similarly, the sample size for Experiment 2 (photometry) was based on the sample size used for reporting local field potential data in our previous papers^16–18^. The data were presented as the group mean ± standard error of the mean (SEM). Statistical analyses were performed with MATLAB and SPSS statistical software (IBM). To determine statistical significance, we used: one-way repeated-measures analysis of variance (ANOVA), two-way mixed/repeated-measures ANOVA, three-way mixed ANOVA, one-sample *t* test, paired and unpaired *t* tests, linear regression analysis, and Pearson correlation. Significance was defined as *p < 0.05, **p < 0.01, ***p < 0.001.

## Results

### Experiment 1—Pharmacological inhibition of the RE impaired acquisition in differential trace eyeblink conditioning

**Figure 1:**
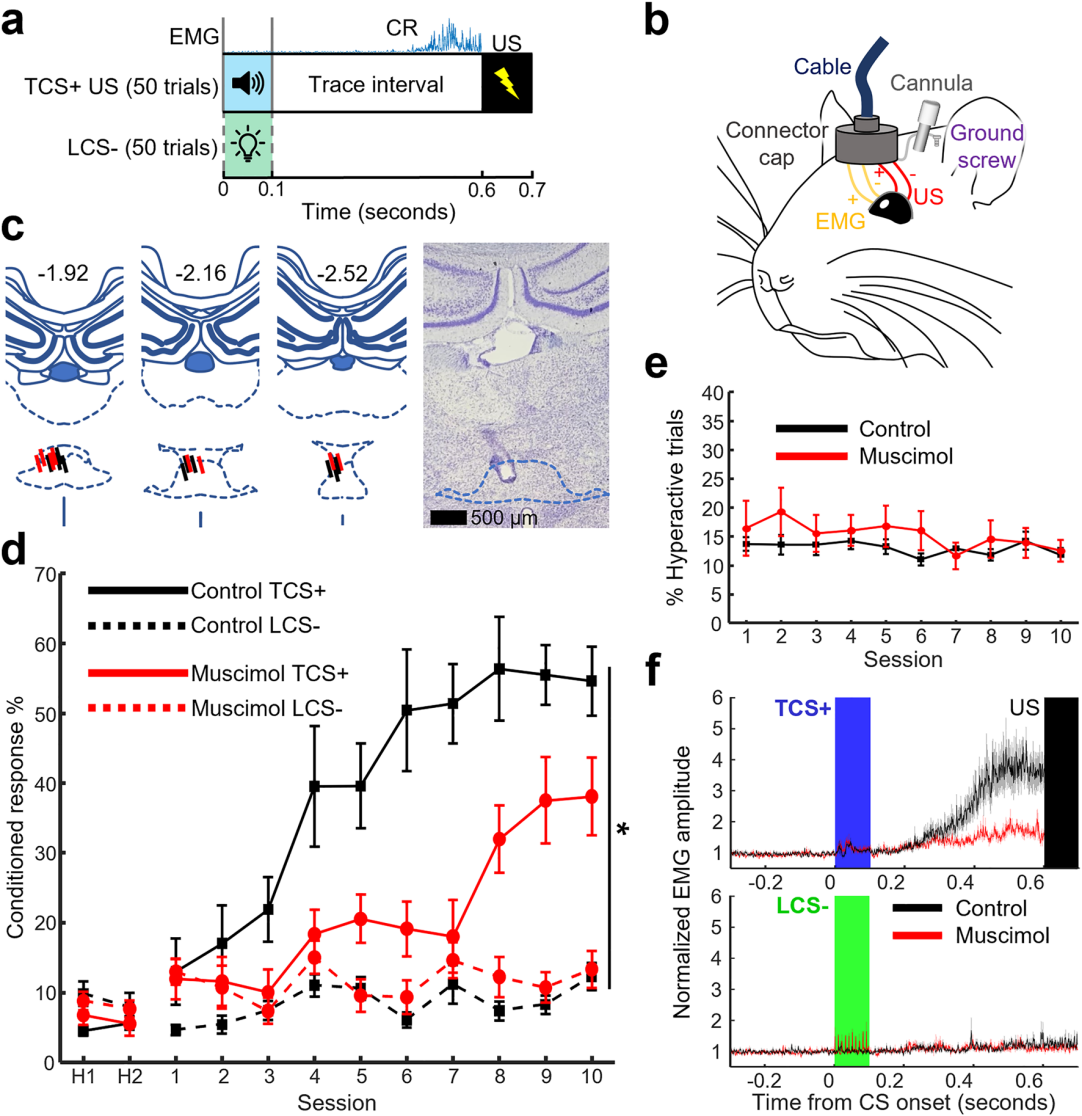
Reversible inactivation of the RE impairs differential associative learning but not stimulus discrimination. (**a**) Behavioral paradigm. The two trial types were randomly intermixed and presented over sessions lasting 50 min. (**b**) Conditioned responses (CR) were detected by recording electromyogram (EMG) activity from the left *orbicularis oculi* (eyelid) muscle. A microinfusion cannula was implanted targeting the RE. (**c**) Left three: histological reconstruction of cannula tip locations in the RE for all rats included in the final analyses. Black and red bars indicate the infusion cannula position for control (N = 9) and muscimol groups (N = 8), respectively. The numbering at the top indicates the anterior–posterior (AP) coordinates from bregma. Right: representative section in the RE. (**d**) Proportion of trials in which rats expressed the CR to the TCS+ and LCS- (*p < 0.05, Group × CS Type interaction). Error bars indicate ± standard error of the mean (SEM). (**e**) Percentage of hyperactive trials (mean ± SEM). (**f**) Averaged normalized EMG amplitude in session 10. Blue and green rectangles indicate TCS+ and LCS- presentation, respectively. A black rectangle indicates the US presentation. Shaded areas indicate ± SEM.

First, we sought to confirm that the RE was necessary for the formation of stimulus associations in DTEBC by examining the impact of pharmacological inactivation of the RE on CR acquisition. Following two days of habituation sessions, 22 rats underwent 10 acquisition sessions, during which the tone stimulus was paired with the US (TCS+), while the light stimulus (LCS-) was presented alone (Fig. 1a). Before each acquisition session, the GABA_A_ receptor agonist muscimol was infused (Fig. 1b) into the RE of 11 rats (muscimol group), whilst the other 11 received infusions of artificial cerebrospinal fluid (control group). Five rats were removed due to mistargeted cannula, leaving eight rats in the muscimol group and nine rats in the control group (Fig. 1c). Over 10 conditioning sessions, the control group gradually increased the proportion of TCS+ trials in which they expressed CRs (Fig. 1d). Compared to controls, the muscimol group expressed CRs in lower numbers of TCS+ trials (Fig. 1d, three-way mixed ANOVA, Session × Group × CS Type interaction, *F*_(9,135)_ = 2.299, *p* = 0.020. Follow-up two-way mixed ANOVA for TCS+ trials, Session, *F*_(9,135)_ = 21.037, *p* < 0.001; Group, *F*_(1,15)_ = 8.266, *p* = 0.012; Session × Group interaction, *F*_(9,135)_ = 3.264, *p* = 0.001). Specifically, the muscimol group expressed significantly lower numbers of CRs on session seven (unpaired *t* tests corrected for multiple comparisons, *t*_15_ = 4.026, *p* = 0.001), whilst there was a trend for lower CR expression on sessions five to six and eight to 10 (*p* = 0.005–0.054). In parallel, both groups showed a small increase in the number of CRs expressed in LCS- trials; however, the frequency of CR expression was greater in the muscimol than control group (follow-up two-way mixed ANOVA for LCS- trials, Session, *F*_(9,135)_ = 2.362, *p* = 0.016; Group, *F*_(1,15)_ = 5.063, *p* = 0.040; Session × Group interaction, *F*_(9,135)_ = 0.930, *p* = 0.502). Furthermore, during the last three days, both groups showed a higher CR% in TCS+ compared to LCS- trials (two-way mixed ANOVA on the averaged CR% across the last three days, Group × CS Type interaction, *F*_(1,15)_ = 8.732, *p* = 0.010; CS Type, *F*_(1,15)_ = 85.170, *p* < 0.001; Group, *F*_(1,15)_ = 4.845, *p* = 0.044). Follow-up unpaired *t* tests corrected for multiple comparisons revealed that the muscimol group had significantly lower CR% in TCS+ trials compared to controls (*t*_15_ = 2.665, *p* = 0.018), whilst CR% in LCS- trials were comparable between groups (*t*_15_ = − 1.421, *p* = 0.176). Additionally, the muscimol group did not differ from the control group in terms of the number of trials during which they showed jumping, rearing, or grooming immediately before CS onset (Fig. 1e, two-way mixed ANOVA, Session × Group interaction, *F*_(9,135)_ = 1.144, *p* = 0.336; Session, *F*_(9,135)_ = 1.765, *p* = 0.081; Group, *F*_(1,15)_ = 0.539, *p* = 0.474), indicating that RE inhibition did not impact baseline activity levels. These findings suggest that RE inhibition impaired rats’ ability to form stimulus associations; however, their ability to discriminate tone from light stimuli was unaffected.

To examine if RE inhibition impacted the temporal pattern of the conditioned eyeblink response, we depicted the averaged integrated CR for both groups and CS types in the last session (Fig. 1f). Compared to the control group, the averaged EMG amplitude in TCS+ trials was lower in the muscimol group, further confirming the reduced number of CRs expressed (Fig. 1f top). However, the temporal patterns of the averaged EMG amplitude were comparable between the two groups. No difference was found between groups for their latency to the onset of the CR (control group: 238 ± 36 ms; muscimol group: 226 ± 41 ms; unpaired *t* test, *t*_15_ = 0.225, *p* = 0.825), nor the latency to the peak (control group: 448 ± 10 ms; muscimol group: 411 ± 17 ms; *t*_15_ = 1.917, *p* = 0.074). Similarly, in LCS- trials, no differences were found between groups for their latency to onset (control group: 192 ± 39 ms; muscimol group: 241 ± 57 ms; *t*_15_ = − 0.723, *p* = 0.481), nor latency to the peak (control group: 434 ± 17 ms; muscimol group: 416 ± 23 ms; *t*_15_ = 0.674, *p* = 0.510). Therefore, although RE inhibition reduced the numbers of CRs expressed, it did not affect the overall temporal pattern of the CRs.

### Experiment 2—Neutral sensory stimuli activate prefrontal terminals within the MD but not RE

**Figure 2:**
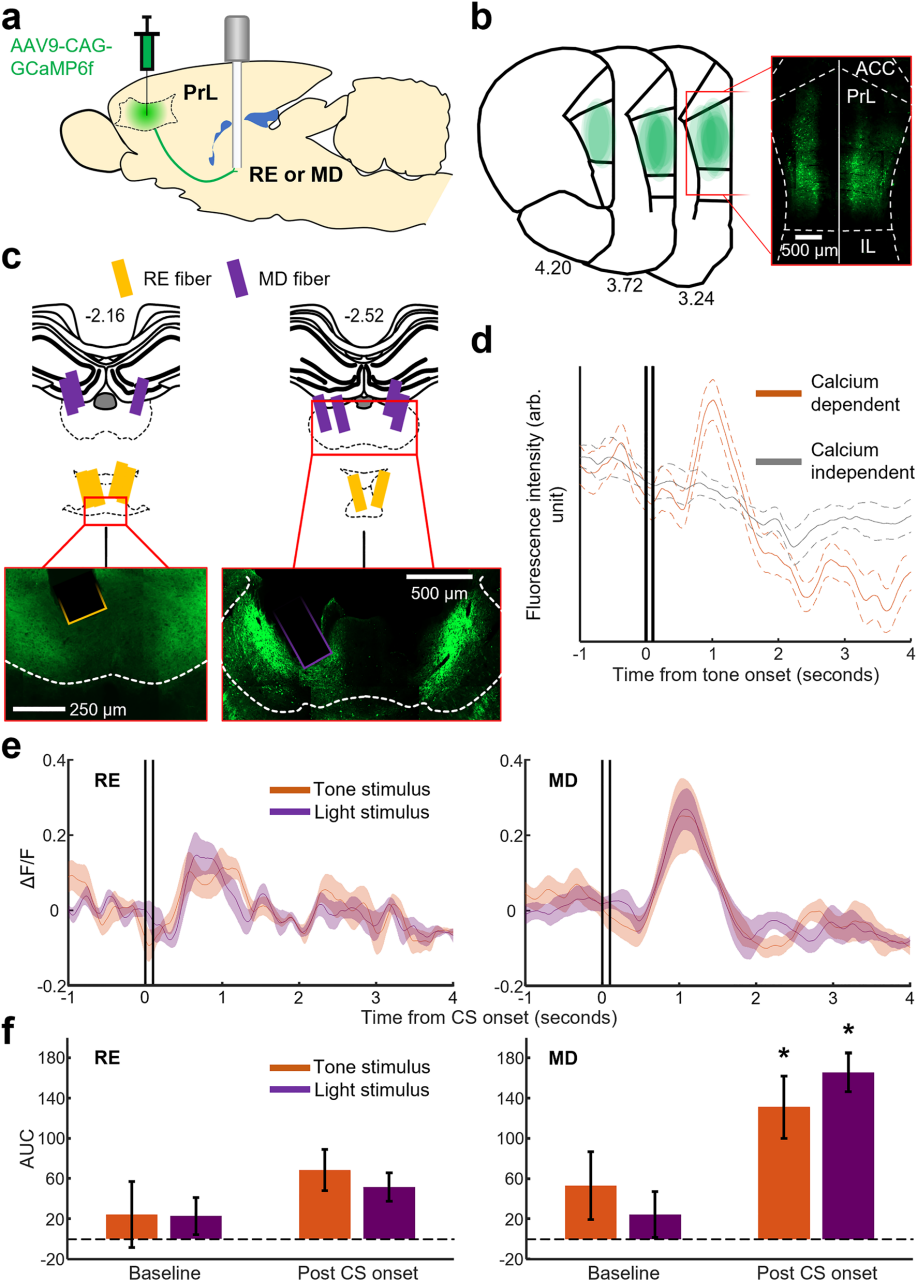
Prefrontal terminals within the RE are not activated by sensory cues lacking mnemonic qualities. (**a**) Prefrontal (prelimbic region [PrL]) terminals expressing GCaMP6f. were recorded within the RE or MD. (**b**) Histological reconstruction and representative section of the mPFC showing GCaMP6f. expression (green). Viral infusions were bilateral; hemispheres were overlaid to display spread in both hemispheres. Numbering indicates the AP coordinate from bregma. Abbreviations: anterior cingulate cortex (ACC), infralimbic cortex (IL). (**c**) Top: histological reconstruction of optic fiber tip placement in the RE and MD. Bottom: representative sections within the magnified area**.** Numbering indicates the AP coordinate from bregma. (**d**) Representative calcium dependent (465 nm) and independent (405 nm) signals from photometric recording of prefrontal terminals within the MD in a representative rat during the presentation of tone. Two vertical lines indicate the onset and termination of the tone. Dashed lines indicate ± SEM (N = 25 presentations). (**e**) Prefrontal terminal responses to stimuli on Session 0 averaged across rats. Shaded areas indicate ± SEM (RE, N = 6 rats; MD, N = 7 rats). (**f**) Session 0 baseline and CS evoked AUC calculated by summation of ΔF/F values in a two second window immediately before and after CS presentations, respectively (*p < 0.05, vs. Baseline). Error bars indicate ± SEM (RE, N = 6 rats; MD, N = 7 rats).

Having established that the integrity of the RE is necessary for the formation of stimulus association, we next investigated how prefrontal terminals within the RE and MD responded to stimuli as rats associated them with the eyelid shock. To this end, we bilaterally infused a viral vector into the mPFC to express the genetically encoded calcium indicator (GCaMP6f.) within mPFC neurons (Fig. 2a). The activity of their terminals within the RE or MD was monitored through a chronically implanted optic fiber in the RE or MD (nine rats for each region). Expression of GCaMP6f. was localized to the prelimbic (PrL) region of the mPFC in both hemispheres, with minimal expression within the neighboring anterior cingulate (ACC) and infralimbic cortices (IL, Fig. 2b). Of the 18 rats, four rats were removed due to mistargeted optic fibers, and one rat was removed due to breakage of the internal fiber core during behavioral conditioning. This left a total of six rats with optic fibers targeting terminals within the RE (RE group) and seven rats with fibers targeting the MD (MD group, Fig. 2c). All subsequent analyses in Experiment 2 were thus performed only on these rats. We confirmed that the 465 nm calcium dependent signal was markedly different from the 405 nm calcium independent signal (Fig. 2d). The ΔF/F values calculated from these two signals allowed us to examine calcium transients minus any contamination from photo-bleaching or motion artifacts (see Methods).

We first investigated the terminal responses to two neutral sensory stimuli prior to conditioning. Following two days of habituation sessions, rats underwent one session of pre-conditioning stimulus testing (Session 0), this involved trials wherein a tone (TS) or light (LS) stimulus was presented alone. During Session 0, presentations of both the TS and LS evoked strong and clearly defined responses from prefrontal terminals within the MD, whilst terminal responses in the RE were weak and harder to distinguish from baseline activity (Fig. 2e). To quantify if these responses were significantly greater than baseline activity, we calculated the area under the curve (AUC) for our ΔF/F values during a two second window before and after CS onset. We found that when compared to baseline activity, terminals within the RE were not significantly activated by presentations of either the TS or LS (Fig. 2f left, two-way repeated measures [RM] ANOVA, Time Period × CS Type interaction, *F*_(1,5)_ = 0.391, *p* = 0.559; Time Period, *F*_(1,5)_ = 1.700, *p* = 0.249; CS Type, *F*_(1,5)_ = 0.112, *p* = 0.751). Conversely, terminals within the MD were strongly activated by both stimuli (Fig. 2f right, two-way RM ANOVA, Time Period × CS Type interaction, *F*_(1,6)_ = 1.112, *p* = 0.332; Time Period, *F*_(1,6)_ = 10.061, *p* = 0.019; CS Type, *F*_(1,6)_ = 0.125, *p* = 0.736). These findings suggest that novel sensory stimuli absent any mnemonic qualities strongly activate prefrontal terminals within the MD but not RE.

### Prefrontal terminals in the RE, but not MD, improve their selectivity for stimulus association with learning

**Figure 3:**
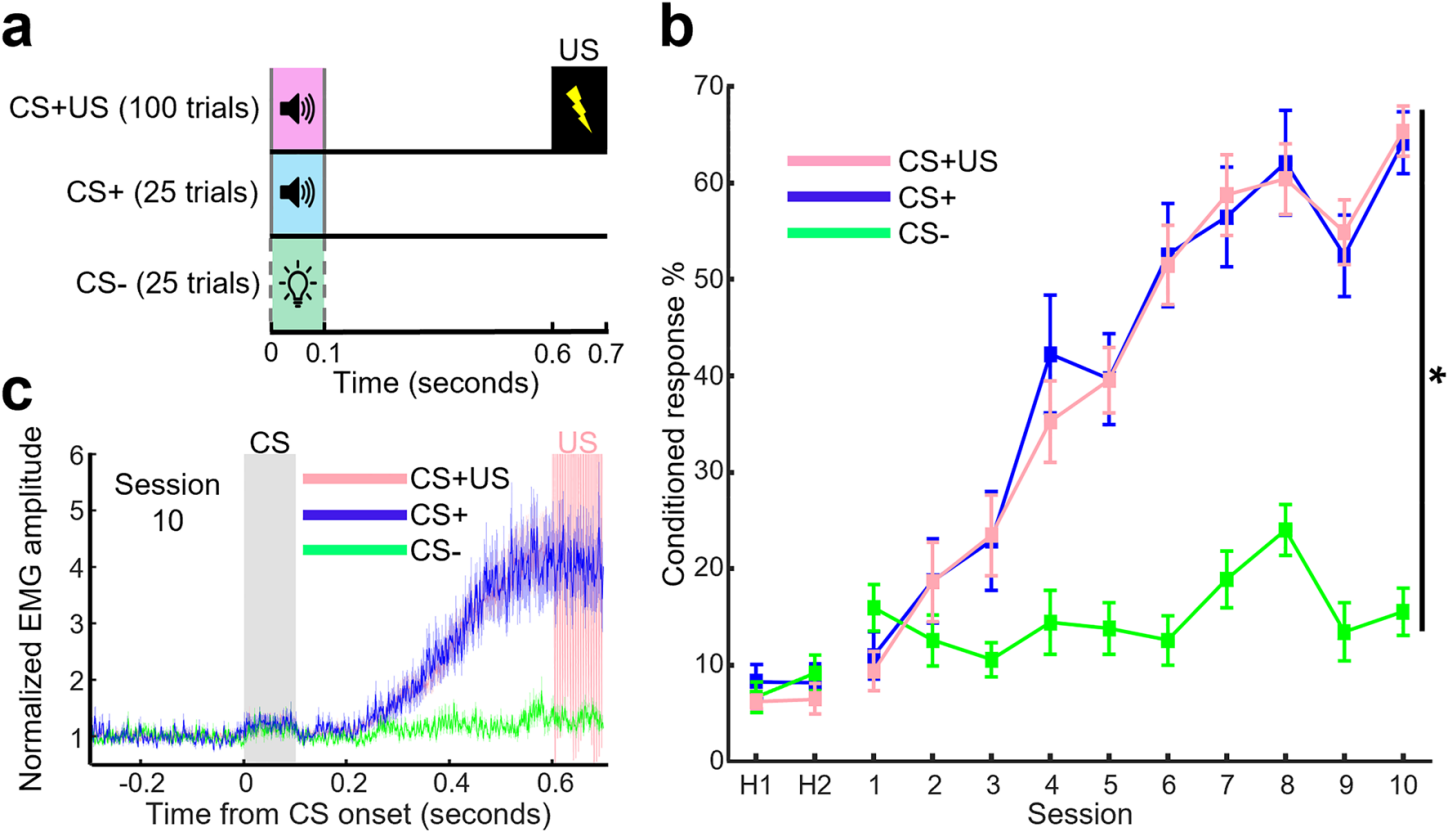
Behavior during photometry recording. (**a**) Behavioral paradigm. The three trial types were randomly intermixed and presented over sessions lasting 75 min. (**b**) Proportion of trials in which rats (N = 13) expressed the CR in each of the three trial types (*p < 0.05, Session × CS Type interaction). Error bars indicate ± SEM. (**c**) Averaged normalized EMG amplitude in session 10. The grey rectangle indicates CS presentation. The high amplitude region of the pink trace (CS+US) represents the artifact caused by the delivery of the US. Shaded areas indicate ± SEM.

Following Session 0, rats were conditioned across 10 days in DTEBC2 (Fig. 3a). The tone was used as the CS+ for 10 rats, five of which had an optic fiber targeting the RE and the other five the MD. The light was used as the CS+ in the remaining one and two rats with an optic fiber in the RE and MD, respectively. Over 10 conditioning sessions, all 13 rats gradually increased the proportion of CS+US and CS+ trials in which they expressed CRs. Whilst the number of CRs in CS- trials remained low (Fig. 3b, two-way RM ANOVA, Session × CS Type interaction, *F*_(18,216)_ = 18.418, *p* < 0.001; Session, *F*_(9,108)_ = 36.220, *p* < 0.001; CS Type, *F*_(2, 24)_ = 72.052, *p* < 0.001). During the last three days the CR% was significantly higher in CS+US and CS+ trials compared to CS- trials (one-way RM ANOVA on the averaged CR% across the last three days, CS Type, *F*_(2,24)_ = 185.663, *p* < 0.001. Paired *t* test corrected for multiple comparisons CS- vs., CS+US: *t*_12_ = 16.541, *p* < 0.001; CS+ : *t*_12_ = 13.524, *p* < 0.001). There was no difference in CR% between CS+US and CS+ trials (paired *t* test corrected for multiple comparisons, *t*_12_ = 0.385, *p* = 0.707). Inspection of the averaged integrated CR for the last day of conditioning revealed similar eyeblink response temporal patterns to that in Experiment 1 (Fig. 3c).

**Figure 4:**
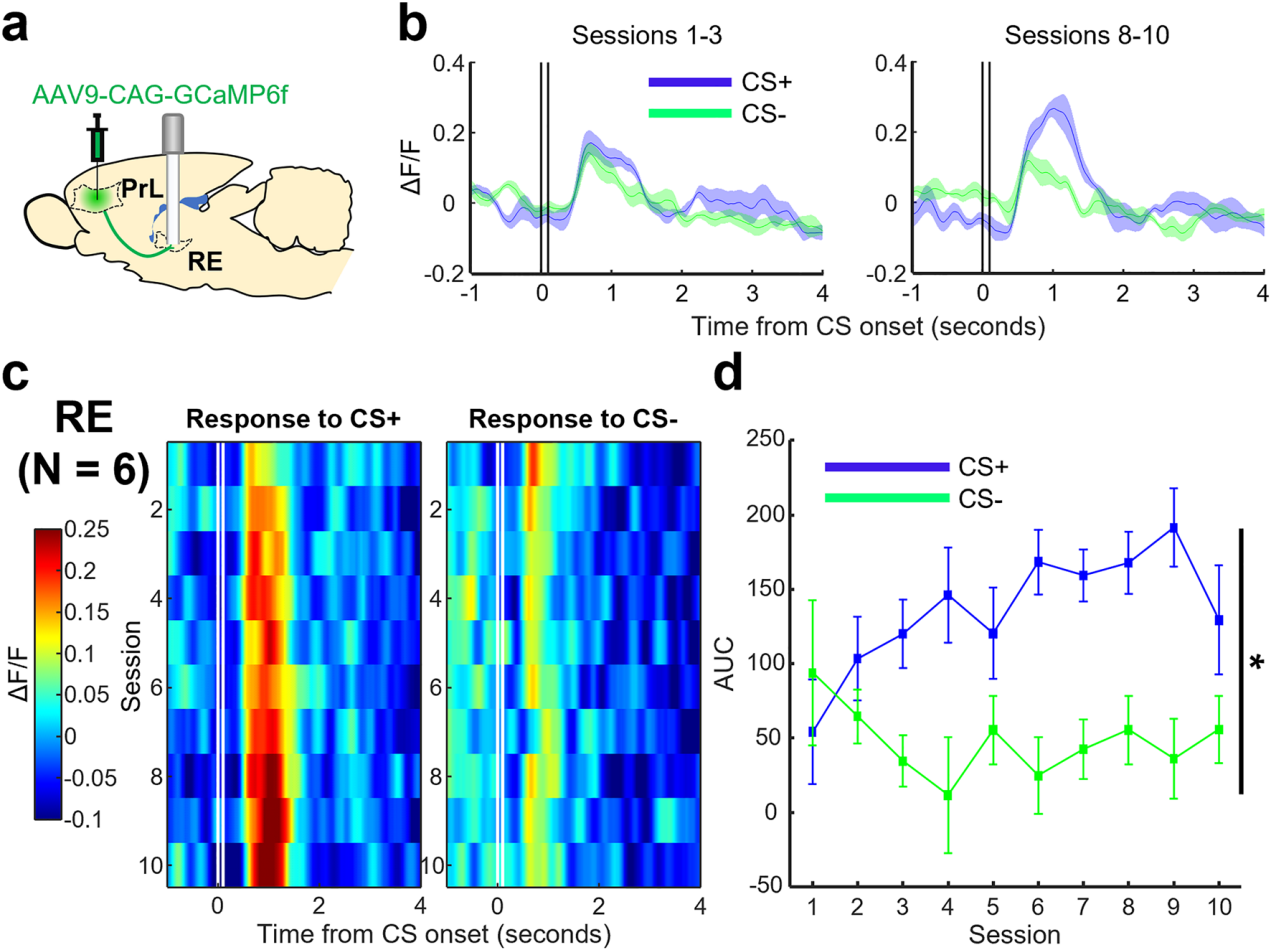
Magnitude of CS+ evoked activity in prefrontal terminals within the RE increases in parallel with the formation of stimulus association. (**a**) Prefrontal (prelimbic region [PrL]) terminals expressing GCaMP6f. were recorded within the RE. (**b**) Left: ΔF/F values averaged across the first three days of conditioning. Right: same as left but for the last three days of conditioning. Vertical black lines represent the onset and termination of the CS. Shaded areas indicate ± SEM. (**c**) Left: averaged ΔF/F values of prefrontal terminals within the RE in response to the CS+ across days of conditioning (y-axis, descending from top to bottom), plotted against time (x-axis). Vertical white lines represent the onset and termination of the CS+ . Right: same as left but for the CS-. (**d**) Averaged AUC in a two second window immediately after CS presentation plotted against days of conditioning (*p < 0.05, Session × CS Type interaction). Error bars indicate ± SEM.

During daily conditioning sessions, we recorded the calcium activity of prefrontal terminals within the RE (Fig. 4a) of six rats. We found that terminals within the RE responded to the CS+ and the CS- at a similar magnitude in the first few sessions (Fig. 4b,c). As conditioning progressed, the CS+ gradually evoked stronger responses; whereas responses to the CS- weakened and then remained low throughout. To better quantify the change in response across days, we calculated AUC by summing ΔF/F values during a two second window right after CS termination. We found that the CS+ evoked response was significantly greater than the CS- (Fig. 4d, two-way RM ANOVA, Session × CS Type interaction, *F*_(9,45)_ = 2.839, *p* = 0.010; Session, *F*_(9,45)_ = 0.695, *p* = 0.709; CS Type, *F*_(1,5)_ = 47.743, *p* = 0.001). Follow-up linear regression analyses revealed that the CS+ evoked responses grew in magnitude across days of conditioning in five out of six RE rats (five rats *p* < 0.05, one rat *p* = 0.411), conversely, no significant effects were seen for CS- evoked responses (six rats *p* > 0.05).

**Figure 5:**
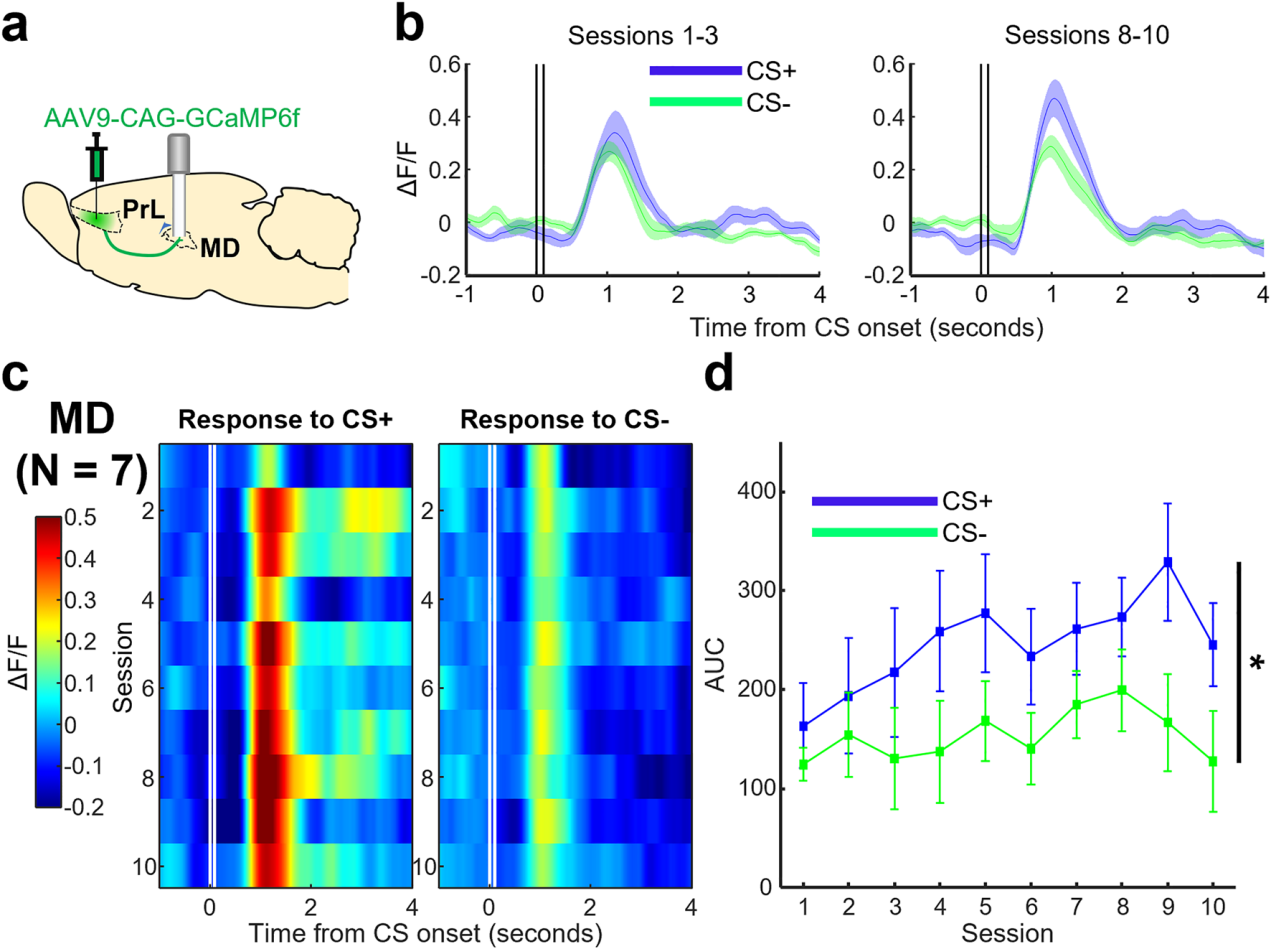
The CS+ evoked greater activity in prefrontal terminals within the MD compared to the CS- starting from the first session of conditioning. (**a**) Prefrontal (PrL region) terminals expressing GCaMP6f. were recorded within the MD. (**b**) Left: ΔF/F values averaged across the first three days of conditioning. Right: same as left but for the last three days of conditioning. Vertical black lines represent the onset and termination of the CS. Shaded areas indicate ± SEM. (**c**) Left: averaged ΔF/F values of prefrontal terminals within the MD in response to the CS+ across days of conditioning (y-axis, descending from top to bottom), plotted against time (x-axis). Vertical white lines represent the onset and termination of the CS+ . Right: same as left but for the CS-. (**d**) Averaged AUC in a two second window immediately after CS presentation plotted against days of conditioning (*p < 0.05, main effect of CS Type). Error bars indicate ± SEM.

In a separate cohort of seven rats, we recorded the calcium activity of prefrontal terminals within the MD during daily conditioning sessions (Fig. 5a). Prefrontal terminals within the MD showed stronger responses to the CS+ than the CS- starting from the first session (Fig. 5b,c). The difference in the response magnitude between the CS+ and CS- appeared starker in subsequent sessions, mostly due to the increased responses to the CS+ . However, these visual impressions were not confirmed by the statistical analysis applied to the AUC values (Fig. 5d, two-way RM ANOVA, Session × CS Type interaction, *F*_(9,54)_ = 0.869, *p* = 0.586; Session, *F*_(9,54)_ = 1.530, *p* = 0.161; CS Type, *F*_(1,6)_ = 9.000, *p* = 0.024). Our findings suggest that although the CS+ evoked greater prefrontal output to both the RE and MD, compared to the CS-, the evolution of this output differed between the two brain regions. Principally, prefrontal output to the RE strengthened across days of conditioning, whilst output to the MD became stronger for the CS+ on the first conditioning day and was maintained stably across subsequent days.

**Figure 6:**
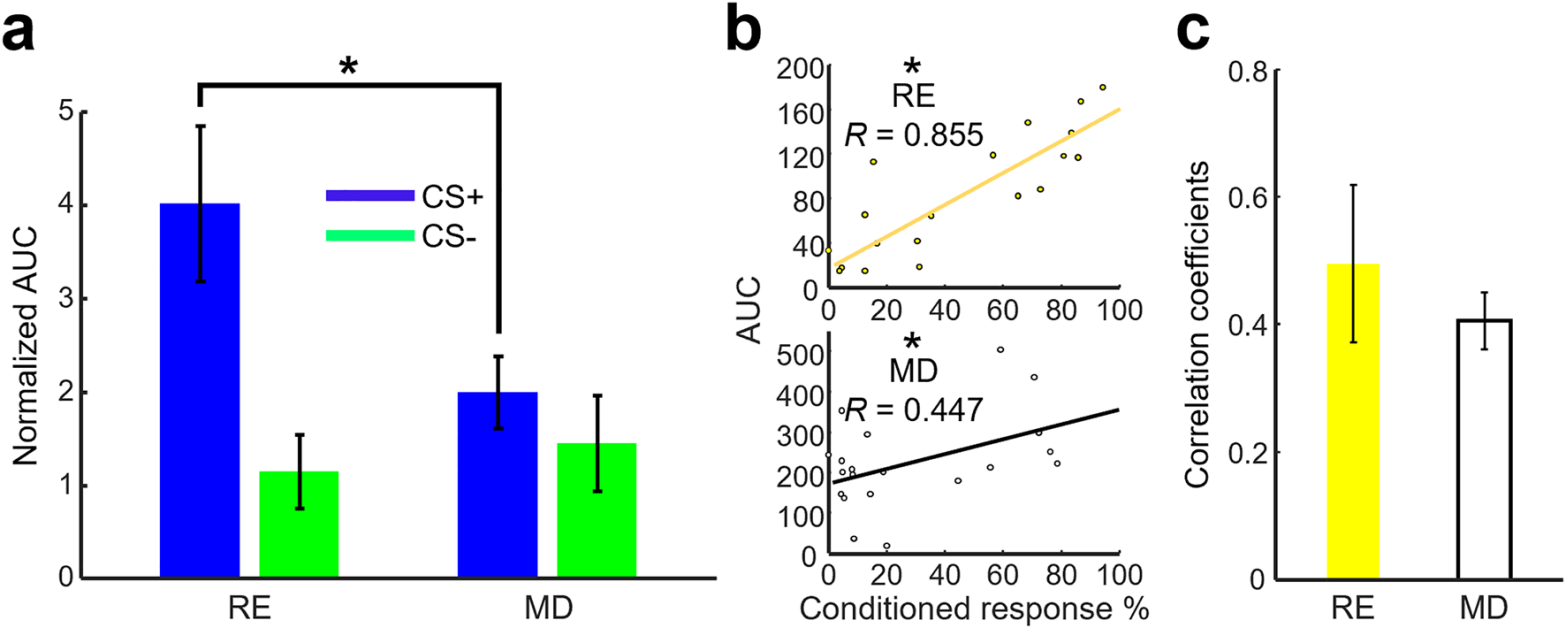
The formation of the CS+US association is accompanied by a stronger increase in CS+ evoked activity in prefrontal terminals within the RE compared to the MD. (**a**) Comparison of CS+ and CS- evoked AUC between prefrontal terminals within the RE (N = 6) and MD (N = 7, *p < 0.05). AUC was averaged during the last three days of conditioning and normalized using Session 0 CS evoked AUC values. Error bars indicate ± SEM. (**b**) Correlations of CR% with CS evoked AUC in two representative rats. Dots within each plot represent the CR% for CS+ or CS- trials plotted against the AUC value for the same trial type across all 10 sessions. Top: RE fiber rat. Bottom: MD fiber rat (*p < 0.05). (**e**) Comparison of averaged correlation coefficients between CR% and AUC (RE, N = 6; MD, N = 7). Error bars indicate ± SEM.

To compare the differences more directly between prefrontal output to the RE and MD, we divided the stimulus-evoked AUC averaged across the last three days of conditioning with the stimulus-evoked AUC prior to conditioning (Session 0). This normalized AUC quantified the relative change of CS evoked terminal activity before and after associative learning. The change in the CS+ evoked response was significantly greater in terminals within the RE than those within the MD (Fig. 6a, two-way mixed ANOVA, Group × CS Type interaction, *F*_(1,11)_ = 4.942, *p* = 0.048; Group, *F*_(1,11)_ = 2.297, *p* = 0.158; CS Type, *F*_(1,11)_ = 10.508, *p* = 0.008. Follow-up unpaired *t* test, *t*_11_ = 2.320, *p* = 0.041). In contrast, the change in CS- evoked responses was comparable between terminals within the RE and MD (*t*_11_ = 0.535, *p* = 0.603). These findings suggest that the formation of stimulus associations resulted in a greater increase of prefrontal output to the RE than to the MD.

To further tighten the relationship between prefrontal thalamic outputs and associative learning, we calculated the Pearson correlation coefficient (r) between AUC values and CR% for both CS types across the 10 sessions of conditioning. Of the six RE rats, three showed a significant (*p* < 0.05) positive correlation between response magnitude and CR% (Fig. 6b). Of the seven MD rats, three showed a significant positive correlation. As a group (Fig. 6c), R-values were significantly different from zero in both RE (one-sample *t* test, *t*_5_ = 3.720, *p* = 0.014) and MD groups (*t*_6_ = 9.056, *p* = 0.001). The R-values were also comparable between RE and MD groups (unpaired *t* test, *t*_11_ = 0.713, *p* = 0.491). Thus, prefrontal outputs to the RE and MD were selective for stimulus associations; however, only the prefrontal outputs to the RE showed a greater improvement in associative selectivity with learning.

## Discussion

Accumulating evidence from behavioral studies has shown that the RE, in particular the mPFC-to-RE pathway, plays an important role in memory formation processes that relies on close communication between the mPFC and HPC^22–24^. However, previous studies did not investigate the degree to which mPFC outputs to the RE are modulated by the sensory and relational features of task stimuli. By monitoring the activity of prefrontal projections terminating in the RE, we found that prefrontal terminal activity became strongly activated by a stimulus only after the stimulus became associated with an aversive outcome. The magnitude of the stimulus-evoked terminal activity was positively correlated with the strength of learned stimulus association. Moreover, the learning-related increase in terminal responses was greater in the RE than in another thalamic output target, the MD. This highlights the unique role of the mPFC-to-RE pathway in routing information related to the behavioral relevance of sensory stimuli during associative learning.

Our photometric recordings demonstrated that prefrontal terminals within the RE were not activated in response to auditory and visual stimuli that were presented by themselves (Session 0; Fig. 2e,f). With learning, however, they became strongly activated by one of the stimuli that were paired with an eyelid shock but not by the other stimulus presented alone (Fig. 4d). These findings suggest that the detected change in mPFC-to-RE output is attributable to associative learning, but not sensitization to repeatedly presented sensory stimuli. Similar differential activity patterns have been previously reported for mPFC oscillatory^17,18^ and spiking activity^12,14,15,50,51^. Furthermore, a manipulation that enhances this differential activity facilitates the formation of stimulus associations^16,17^. Collectively, these findings suggest that the mPFC plays a critical role in detecting and encoding relevant stimulus associations^52^. The present findings extend this notion by demonstrating that the detected relevance is routed to the RE.

When the RE was pharmacologically inactivated, rats were unable to form the association between the stimulus and the aversive outcome but did not increase their responses to the other stimulus presented alone (Fig. 1d). These patterns indicate that the RE is necessary for the formation of stimulus associations, but not for sensory discrimination. The present observation is consistent with previous reports that RE perturbation via either muscimol^53^ or high frequency electrical stimulation^40^ impaired memory acquisition in trace fear and trace eyeblink conditioning, respectively. At the same time, even with RE inactivation, rats still showed a small degree of associative learning. This is likely the result of incomplete RE inactivation, as the RE extends far across the anterior–posterior axis, which is beyond the spread of our infused muscimol.

Given the selectivity of prefrontal projections in the RE for the behavioral relevance of sensory events, the observed learning deficits following RE inhibition could be due to the deprivation of the HPC from the mPFC relevancy signal. Alternatively, RE inhibition could also disrupt the synchronization of neural activity between the mPFC and HPC, which is known to play an essential role in temporal associative learning^19,54^. In urethane-anesthetized rats, RE inactivation disrupted both the temporal patterning of gamma bursting (GB) and its synchronicity between the mPFC and CA1; however, the frequency of these GB occurrences in both regions was unaffected^30^. More recently, we have found that the incidence rate of mPFC GB during trace intervals in DTEBC was positively correlated with task acquisition^18^. If similar GB also occurs in CA1 at the same time, RE inhibition might disrupt their synchrony, leading to disrupted mPFC-HPC communication. Additionally, as we did not selectively inhibit mPFC-to-RE projections, the impaired learning with RE inhibition may be due to the disruption of information transfer and/or modulatory effects from the dorsal HPC to mPFC via the RE.

In parallel to its role in information gating, several works using contextual fear conditioning tasks argued that the RE also controls the precision of contextual fear memories^35–38^. Specifically, inactivation of mPFC output to the RE or direct silencing of RE projections led to the acquisition of a fear memory that lacked selectivity for the original conditioning context^35^. Notably, imprecise contextual memories acquired during periods when the RE was inactivated are acquired independently of the hippocampus^36^. In contextual fear conditioning, subjects with reduced hippocampal function form an elemental association of the footshock with a simple environmental cue but not with detailed representations of the conditioning environment, resulting in the loss of context-specificity^55,56^. Therefore, the apparent role of RE in controlling memory precision may be one manifestation of its more general role in modulating the engagement of the HPC in memory encoding^36^. This idea fits well with the observed learning deficits in our task. Specifically, in TEBC, the HPC is required for bridging the temporal gap between the CS and US, which cannot be circumvented by engaging a low-level learning strategy^1–3^. Thus, the RE inhibition resulted in impaired CR acquisition (Fig. 1d) because it could not adequately engage the HPC during conditioning. Our photometry data further extended this idea by suggesting that this functionality of the RE is controlled by the mPFC output signaling the relevance of ongoing events.

The MD is reciprocally connected with the mPFC^41–43^ but lacks projections to and from the HPC^44,45^. This anatomical feature led us to contrast prefrontal outputs between the MD and RE, uncovering several qualities unique to the mPFC projections to the MD. First, prefrontal terminals in the MD were strongly activated by sensory stimuli before the conditioning began. Second, once conditioning began, prefrontal outputs to the MD differentiated between CS+ and CS- before rats developed differential CRs (Fig. 3b and 5d). Additionally, the response magnitude did not increase across subsequent conditioning sessions. Based on these findings, we argue that the loop between the mPFC and MD helps sustain stimulus representations within the mPFC during stimulus-outcome intervals, as proposed in various forms of working memory tasks^45,57–62^. For example, MD terminals within the mPFC are necessary for the sustained firing of mPFC neurons during the delay period of a delayed non-match to sample T-maze task^57^. Additionally, in another WM task requiring the maintenance of a sensory cue over a delay period for a subsequent rule-based choice selection, delay period MD activity was found to be dependent on inputs from cue-selective mPFC neurons^58,61^. These studies highlight the importance of MD projections to the mPFC in sustaining behaviorally relevant information and the MD’s dependence on stimulus evoked mPFC output to initiate this process. Based on this, novel or possibly relevant stimuli should elicit mPFC output to the MD, as the animal would need to hold the stimulus in an active state to assess whether it is behaviorally relevant enough to warrant its commitment to memory. This view also corroborates with findings from studies wherein rabbits with MD lesions required a greater number of conditioning sessions to form the stimulus association compared to non-lesioned controls in TEBC^63^, whilst performance in delay eyeblink conditioning, which lacks a trace interval, is only minimally, if at all, affected^64,65^.

## Conclusion

Our findings here identified functional dissociations between two major prefrontal-thalamic pathways by uncovering the learning-induced increase in prefrontal output to the RE, indicating that the information outputted to the RE signals for the relational features of sensory stimuli. Future studies will need to investigate what kind of information is sent from the RE to the HPC and how it affects hippocampal neural processing supporting temporal associative learning.

## Data Availability

All data generated and analyzed in this study are available from the corresponding author upon reasonable request.
